# Spectral X-ray dark-field signal characterization from dual-energy projection phase-stepping data with a Talbot-Lau interferometer

**DOI:** 10.1038/s41598-022-27155-1

**Published:** 2023-01-14

**Authors:** Kirsten Taphorn, Lennard Kaster, Thorsten Sellerer, Alexander Hötger, Julia Herzen

**Affiliations:** 1 Research Group Biomedical imaging Physics, Department of Physics, School of Natural Sciences, Technical University of Munich, 85748 Garching, Germany; 2 Munich Institute of Biomedical Engineering (MIBE), Technical University of Munich, 85748 Garching, Germany; 3 Walter Schottky Institute and Physics Department, School of Natural Sciences, Technical University of Munich, 85748 Garching, Germany; 4 Munich Center for Quantum Science and Technology (MCQST), 80799 Munich, Germany

**Keywords:** Imaging techniques, X-rays

## Abstract

Material-selective analysis of spectral X-ray imaging data requires prior knowledge of the energy dependence of the observed signal. Contrary to conventional X-ray imaging, where the material-specific attenuation coefficient is usually precisely known, the linear diffusion coefficient of the X-ray dark-field contrast does not only depend on the material and its microstructure, but also on the setup geometry and is difficult to access. Here, we present an optimization approach to retrieve the energy dependence of the X-ray dark-field signal quantitatively on the example of closed-cell foams from projection data without the need for additional hardware to a standard grating-based X-ray dark-field imaging setup. A model for the visibility is used to determine the linear diffusion coefficient with a least-squares optimization. The comparison of the results to spectrometer measurements of the linear diffusion coefficient suggests the proposed method to provide a good estimate for the energydependent dark-field signal.

## Introduction

Acquiring data with different photon spectra enables access to advanced material-specific information, by exploiting the differences in the energy-dependent attenuation. For example, diagnostic imaging benefits from the ability to differentiate coagulated blood and iodine contrast agents^1^ and to determine effective atomic numbers^2^. Furthermore, the reduction of beam-hardening artifacts in virtual mono-energetic X-ray images^3^ was demonstrated to provide additional benefits to conventional X-ray imaging.

In recent years, various imaging techniques have been developed that exploit additional contrast channels besides the conventional attenuation^4–7^. Within this work, we focus on grating-based X-ray dark-field (XDF) imaging with a Talbot-Lau interferometer^8^. Additionally to the conventional attenuation image, a dark-field image based on ultra small-angle scattering is retrieved. By that, information about the microstructure of the sample is obtained without having to resolve it directly^8^. The shift in phase of a wavefront, induced by passing through the sample, generates a third contrast channel, the so-called phase-contrast^9^.

More and more potential applications of X-ray dark-field imaging are being investigated. In terms of medical diagnostics, dark-field imaging was demonstrated to enable the detection of early-stage lung diseases^10,11^. The transition to clinical routine is currently taking place with the first patient scanner capable to provide dark-field radiography images of a living human thorax^12^. Alternative applications range from foreign body detection^13,14^ to mammography^15,16^. Moreover, the X-ray dark-field signal induced by sub-pixel microstructures is of interest for material science. For instance, access to the samples' porosity is enabled without the requirement of directly resolving small cavities in the sample^17^.

Following these achievements, the next step is to combine spectral and dark-field imaging. In first studies, we have demonstrated that for instance materials with different sub-pixel microstructure can not only be differentiated, but also qualitatively ranged regarding their microstructure size with spectral X-ray dark-field imaging^18^, followed by quantitative material decomposition which we successfully transferred from the absorption channel to the dark-field channel^19^. Latest, we proposed an application of spectral X-ray dark-field imaging for thorax radiography, where a direct differentiation of pathological changes, such as emphysema and fibrosis, in the human lung parenchyma was enabled with spectral X-ray dark-field imaging, which is not possible in single-spectrum conventional dark-field radiography^20^.

Aiming for material-selective information, analysis methods for dual-energy data require either a calibration routine^19^ or prior knowledge of the behavior of the observed signal with the X-ray energy. In the case of the attenuation contrast, the attenuation coefficient is a well-known material-specific property and can be found in several databases (e.g. the XMuDat database^21^). For the dark-field channel, however, the linear diffusion coefficient, defined analogously to the attenuation coefficient, strongly depends on the material and its microstructure^22^, as well as the setup geometry^23,24^ and is thus, not a simple material-specific quantity. Obtaining the linear diffusion coefficient precisely requires either micro computed tomography data sets with sufficient resolution (i.e. ideally below one micrometer) and subsequent analysis of the microstructure resolved therein, or measurements with spectrometers.

In this work, we present an optimization approach to estimate the linear diffusion coefficient of a scatterer from simple phase-stepping projection data. By comparing a set of dual-energy X-ray dark-field projections of a scattering material to a model function for the expected visibility, the quantitative linear diffusion coefficient, approximated by a power-law, is retrieved via a least-squares optimization. The results are the energy dependence as well as the strength of the material’s dark-field signal. Here, we demonstrate the proposed approach in experiments and validate the quantitative optimization results with spectrometer measurements.

## Material and methods

### Optimization model

The visibility *V* represents the ratio of the amplitude and the mean value of the sinusoidal stepping curve acquired during a phase-stepping scan^8^. The dark-field signal *D* is related to the visibility reduction by the sample, given as the ratio of the visibilities measured with ($V_{S}(E)$) and without sample (*V*(*E*)),

1 $$\begin{aligned} \frac{V_{S}(E)}{V(E)}=e^{- \varepsilon (E)\cdot t}. \end{aligned}$$

Here, the sample with a path length *t* in projection direction is assumed to be homogeneous. The linear diffusion coefficient $\varepsilon (E)$ is defined as^24^,

2 $$\begin{aligned} \varepsilon (E)= \sigma (E)\cdot \left[ 1-G(\xi _\mathrm{corr}(E) )\right] . \end{aligned}$$

The scattering cross-section is denoted with $\sigma (E)$ with an energy dependence of $\sigma (E) \propto E^{-2}$^23^. The normalized projection $G(\xi _\mathrm{corr}(E))$ of the autocorrelation function of the sample’s real-space electron density distribution (referring to the microstructure of the sample)^24,25^ is sampled at the correlation length $\xi _\mathrm{corr}$ of the Talbot-Lau interferometer (referring to the setup geometry)^24^, which is given by

3 $$\begin{aligned} \xi _\mathrm{corr}(E)=S\cdot \lambda (E), \end{aligned}$$

and decreases with increasing X-ray energy ($\lambda =hc/E$). The sensitivity *S* of the Talbot-Lau interferometer is separated into a setup-specific factor and a factor depending on the position of the sample in the setup^26^, and can be simplified to

4 $$\begin{aligned} S= {\left\{ \begin{array}{ll} \frac{d_{\mathrm{Sample},G2}}{p_{2}}, &{} \text {for the sample positioned between}\, G_1\, \text {and}\, G_2, \\ \frac{d_{G0,\mathrm{Sample}}}{p_{0}}, &{} \text {for the sample positioned}\, G_0\, \text {and}\, G_1. \end{array}\right. } \end{aligned}$$

Depending on the position of the sample, the grating period *p* of either $G_0$ or $G_2$ is required, as well as the distance *d* between the sample and the respective grating.

If the correlation length is much larger compared to the microstructure of the material, the energy dependence of $\varepsilon$ is only given by the scattering cross-section $\varepsilon (E) \propto E^{-2}$. For correlation lengths smaller compared to the microstructure, the energy dependence changes because $G(\xi _\mathrm{corr}(E) ) \ne 0$. The linear diffusion coefficient can be approximated by a power-law

5 $$\begin{aligned} \varepsilon (E) \approx \frac{a}{E^b}, \end{aligned}$$

where *a* gives the signal strength and *b* determines the energy dependence of the dark-field signal. The derivation of this approximation can be found in^19^. Although the power-law is derived for spheres, it was already demonstrated in simulation^20^ and experiment^19^ that this assumption also applies to complex homogeneous microstructures with no long-range order.

When imaging with a polychromatic X-ray spectrum, the visibility in a phase-stepping scan of a scattering material with thickness $t_i$ can be modeled by

6 $$\begin{aligned} \hat{V_i^s}=\int D^s (E) \cdot V(E)\cdot \mathrm{exp}\left( - \frac{a}{E^b} \cdot t_i \right) dE. \end{aligned}$$

The visibility spectrum is denoted with *V*(*E*). The normalized detected spectrum $D^s (E)$ results from the X-ray source spectrum which gets attenuated by all components in the beam, such as gratings and filters, $\Phi ^s (E)$, multiplied by the quantum efficiency $\eta (E)$ of the sensor layer. To model an integrating detector, the spectrum has to be weighted with the energy *E*,

7 $$\begin{aligned} D^s (E)=\frac{\Phi ^s (E) \cdot \eta (E) \cdot E}{\sum _E \Phi ^s (E) \cdot \eta (E) \cdot E }. \end{aligned}$$

For different thicknesses $t_i$ of a material measured with two different photon spectra *s*, the difference between the measured and the modeled visibility, $V_i^s$ and $\hat{V_i^s}$, respectively,

8 $$\begin{aligned} L(a,b)=\sum _s \sum _i \left( V_i^s - c_s \cdot \hat{V_i^s} \right) ^2, \end{aligned}$$

is minimized. To keep the flat-field visibility consistent in the optimization model, the expected visibility $\hat{V^s_{0}}$ for $t=0$ mm (calculated with Eq. 6) was factorized for low and high energy measurements individually, such that it equals the measured flat-field visibility $V^{s}_{0}$,

9 $$\begin{aligned} c_s=\frac{V^{s}_{0}}{\hat{V^s_{0}}}. \end{aligned}$$

The unweighted and non-linear least-squares problem in Eq. (8) was solved with the Nelder-Mead algorithm^27^ to retrieve parameters *a* and *b* of the linear diffusion coefficient.

### Experiments

#### Sample materials

The phantom materials were weakly absorbing closed-cell structural foams made from polymethacrylimide (Evonik Industries AG, Essen, Germany), which are commonly used in aerospace and automotive industries as cores for sandwich constructions. In Table 1 the phantom materials are listed. The different names indicate different averaged cell sizes, the number gives the density in kg/$\hbox {m}^3$. The visually estimated cell size for RIMA 71 is lower compared to IGF 71.

**Table 1:** Sample materials and corresponding thicknesses of the step phantoms.

Material	Density [kg/$\hbox {m}^3$]	Step phantom thicknesses [mm]
RIMA 71	71	10, 20, 30
IGF 71	71	10, 20, 30

A step phantom from each material was build with a thickness ranging from 10 mm up to 30 mm in 10 mm steps.

For a visual comparison of the materials, helium-ion microscopy images were taken with a ZEISS Orion NanoFab. The beam current was set to be 0.46 pA with a dwell time of $20\,\upmu$s for IGF 71. The image of RIMA 71 was taken with a beam current of 0.60 pA and a dwell time of $10\,\upmu$s. The acceleration voltage was 30 kV with an aperture of 10 $\upmu$m for both images. Additionally, electrons were accelerated by the flood-gun, in order to compensate the positive charges accumulating upon helium-ion irradiation. This ensured a better image quality, by preventing the distortion of the focused ion beam.

#### Setup, data acquisition and processing

**Figure 1:**
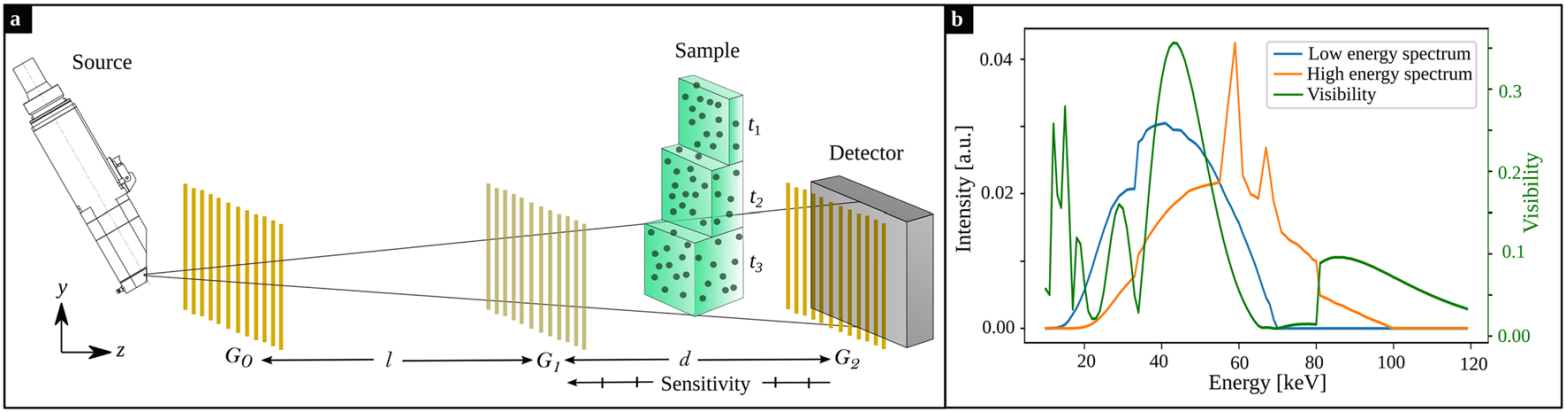
Experimental setup, source spectra and visibility spectrum. (**a**) The Talbot-Lau interferometer consists of three gratings. The sample is a step phantom consisting of different thicknesses of the material of interest. The sensitivity increases towards $G_1$. (**b**) The simulated detected spectra (cf. Eq. 7) are plotted in blue and orange for the low and high energy bin, respectively. The simulated visibility spectrum for the given grating parameters and setup geometry is shown in green.

In Fig. 1a the imaging setup is sketched. The X-ray source is a XWT-160-SE microfocus tube (Xray WorX, Garbsen, Germany) with a tungsten anode and a 2 mm tube window made from beryllium. The Talbot-Lau interferometer consists of three gratings. The source grating $G_0$ and analyzer grating $G_2$ are absorption gratings made from gold with a height of $180\,\upmu \text {m}$, a period of $6.0\,\upmu \text {m}$ and a duty cycle of 0.55. The reference grating $G_1$ is a $\pi$-shifting phase grating made from gold with a height of $8.6\,\upmu \text {m}$, a period of $6.0\,\upmu \text {m}$ and a duty cycle of 0.5. The design energy of the grating interferometer is 45 keV. The Talbot-Lau interferometer is build in symmetrical alignment in the third Talbot order. The distance between the gratings is $l=d=97.9$ cm. For a homogeneous horizontal visibility, all gratings are bend with a radius corresponding to their source-grating distance each. A conventional flat-panel detector (PaxScan4030CB, Varex Imaging, Salt Lake City, Utah) was used, with a pixel size of $194\,\upmu \text {m}$ and a $600\,\upmu \text {m}$ thick caesium iodide scintillation layer.

Dual-energy X-ray dark-field data was acquired with two different photon spectra. For the low energy spectra the acceleration voltage was $70\,$kV at 60 W. For the high energy spectrum an acceleration voltage of $100\,$kV and 70 W was used and the spectrum was filtered with $3.00\,$mm aluminum.

The sample was placed between $G_1$ and $G_2$ at 10 different sensitivities (different positions along the *z*-axis in Fig. 1a) between $S=70\cdot 10^3$ and $S=115\cdot 10^3$ in steps of $\Delta S=4.5\cdot 10^3$ (according to Eq. 4; correlation lengths according to Eq. 3 for the design energy: 1.92 $\upmu$m to 3.16 $\upmu$m in steps of $\Delta \xi _\mathrm{corr}\approx$ 0.13 $\upmu$m). At each position, every thickness of the step phantoms of all materials were measured with a phase-stepping. Prior, a flat-field was acquired at each sensitivity position repeatedly. The $G_0$ was stepped over one grating period with 7 phase steps. The exposure time for every step was $1\,$s. Signal extraction was performed pixel-wise.

Because the spectra are part of the model function for the expected visibility in Eq. (6), both low and high energy spectrum as well as the visibility spectrum are required. Figure 1b shows the source spectra for low and high energy bin in blue and orange, respectively, simulated using the TASMIP algorithm^28^. The visibility spectrum of the Talbot-Lau interferometer is independent of the tube voltage and was simulated for monochromatic X-rays ranging from 10 to 150 keV with a wave-optical simulation package, where the free-space propagation is implemented according to the Fresnel scaling theorem^29^. For the given grating parameters and setup geometry, the simulated visibility spectrum is plotted in green in Fig. 1b.

For a comparison of the proposed optimization approach to the ground truth of the linear diffusion coefficient, the energy dependent dark-field signal of every material was measured with a spectrometer (Amptek Inc., Bedford, Massachusetts) at the same grating interferometer. The spectrometer has a cadmium-telluride sensor layer with a thickness of $1\,$mm and has 2048 energy bins, ranging from 0 to 160 keV for the used gain settings. For the lowest and highest sensitivity ($S=70 \cdot 10^3$ and $S=115 \cdot 10^3$, respectively), the dark-field signal was measured with a phase stepping with 7 phase steps of both materials with a thickness of 10 mm. The acceleration voltage was 120 kV at 11 W. The exposure time was 200 s per phase step to achieve sufficient statistics. The measured spectra were corrected for escape peaks as well as the efficiency of the sensor layer using the XRS-FP correction software (Amptek Inc., Bedford, Massachusetts).

The energy dependent visibility from the spectrometer measurements were extracted for each energy bin individually. Dividing the visibility spectrum from the sample scan by the flat-field reference provided the energy dependent dark-field signal.

## Results

### Optimization results

**Figure 2:**
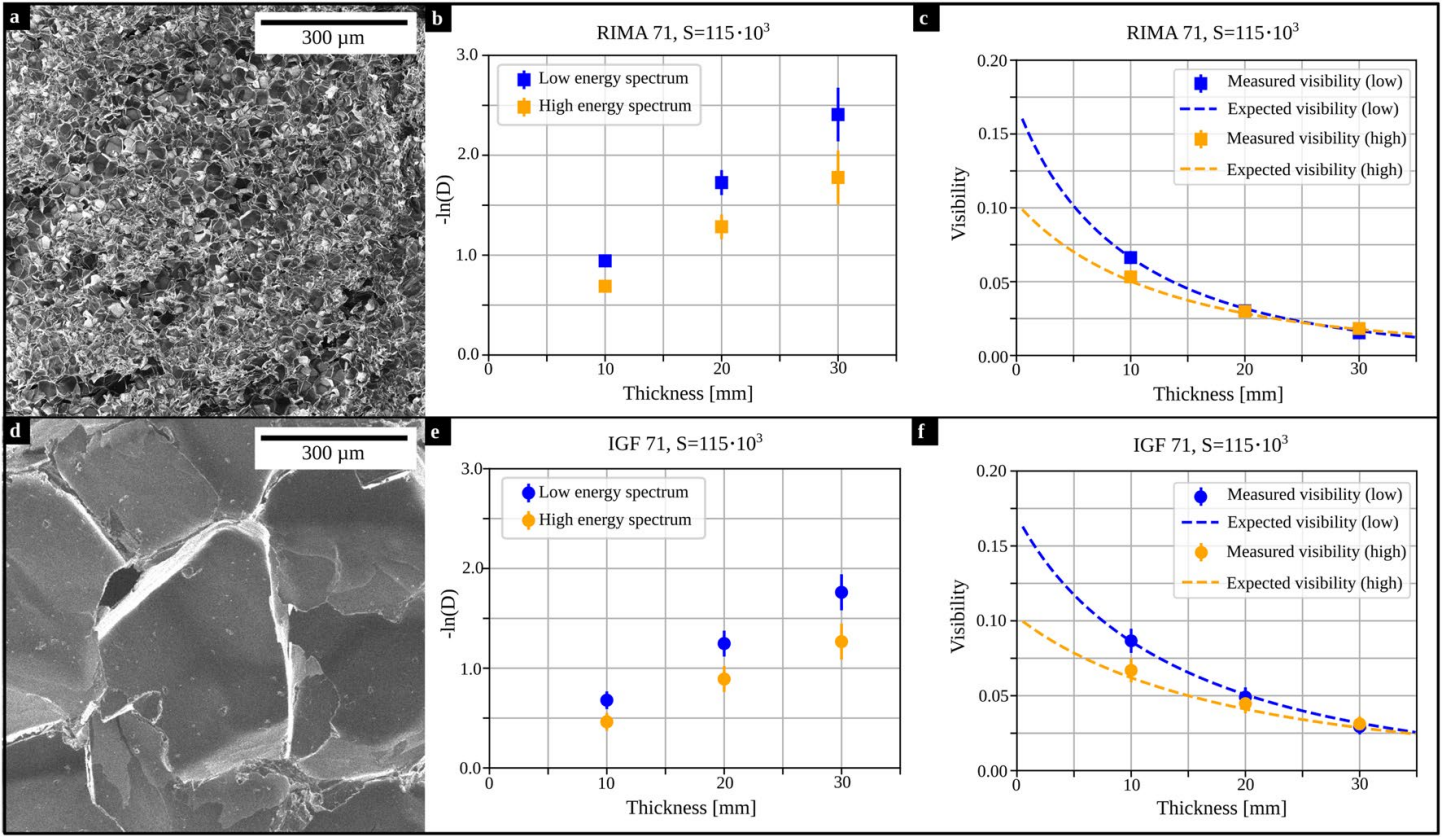
Required data and results from the optimization. (**a**,**d**) Helium-ion microscopy images of RIMA 71 and IGF 71, respectively. (**b**,**e**) Measured dark-field signals for increasing thicknesses of the materials. The dark-field values vary for both materials. (**c**,**f**) Comparison between measured (data points; standard deviation given as error bars) and expected visibility (dashed line) calculated for the optimized parameters *a* and *b* and Eq. (6), at $S=115 \cdot 10^3$.

Figure 2a,d show Helium-ion microscopy images of RIMA 71 and IGF 71, respectively, which demonstrate a significant difference in the size of their microstructure. As expected from Eq. (1), their measured dark-field signal increases for increasing sample thicknesses, depicted in Fig. 2b,e for the highest sensitivity investigated ($S=115\cdot 10^3$). Because the linear diffusion coefficient is inverse proportional to the X-ray energy, its cross-section for high energies is lower compared to low X-ray energies. Therefore, the dark-field signal in the high energy bin is smaller compared to the low energy bin.

In Fig. 2c,f, the measured visibility values are depicted as squares and dots representing RIMA 71 and IGF 71, respectively. The measured visibility is the mean visibility from the sample scans in a region-of-interest in the centre of the field-of-view. The expected visibility with the optimized parameters *a* and *b* (RIMA 71: $a=24517$ /mm, $b=3.32$; IGF 71: $a=36207$ /mm, $b=3.52$) was calculated with Eq. (6) and is illustrated with dashed lines for low and high energy bin, in blue and orange, respectively. For both materials, the expected visibility fits the measured data well.

**Figure 3:**
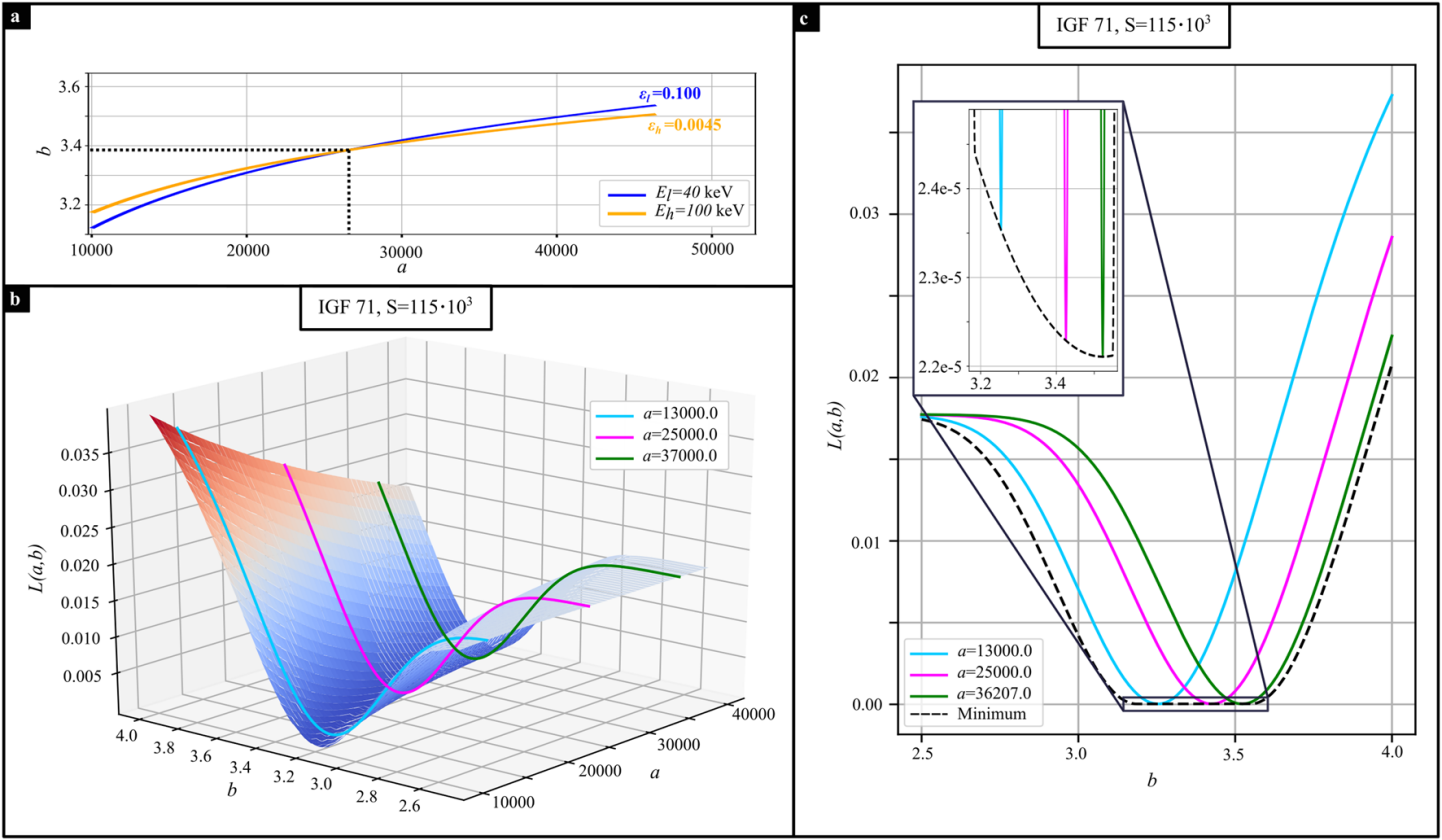
Shape of the optimization landscape. (**a**) Different combinations to achieve $\varepsilon _l$ and $\varepsilon _h$, for different energies $E_l$ and $E_h$, with an intersection, marked with black dotted lines. (**b**) Optimization landscape for IGF 71 at $S=115\cdot 10^3$. (**c**) Line plots through the landscape as indicated in (**b**). The black dotted line provides the minimum of *L*(*a*, *b*) depending on *b*. The zoom-in visualizes the global minimum at $b=3.52$ and $a=36207$ /mm.

Performing the optimization approach for one spectrum only would not provide a unique solution for the optimization, even for multiple thicknesses $t_i$. Assuming a linear diffusion coefficient of $\varepsilon (E_l)=0.1$ for an energy of $E_l=40\,$keV for a fixed sample thickness, and a second measurement with different energy ($E_h=100\,$keV with $\varepsilon (E_h)=0.0045$), then there is a unique combination of *a* and *b* that describes both measurements simultaneously (cf. Fig. 3a).

The deviation value *L*(*a*, *b*) calculated from Eq. (8) is plotted in Fig. 3b for IGF 71 for a sensitivity of $S=115\cdot 10^{3}$ (corresponding measurements: Fig. 2e,f). The optimization landscape has a channel along the *a*-axis. Line plots through the optimization landscape are depicted in Fig. 3c for the positions indicated in Fig. 3b. The black dotted line in Fig. 3c depicts the minimum of *L*(*a*, *b*) for every *b*. The zoom-in shows, that a global minimum of *L*(*a*, *b*) is present at $b=3.52$ (for $a=36207$ /mm). For smaller and larger *b*, *L*(*a*, *b*) increases because the combinations of *a* and *b* to provide the measured linear diffusion coefficient differ more and more between the two spectra (cf. Fig 3a, where the blue and orange lines diverge further apart, as one moves away from the point of intersection). For parameter *a* the same plot as in Fig. 3c can be made. This demonstrates that the proposed optimization method for the linear diffusion coefficient has a unique solution.

### Comparison with spectrometer measurements

**Figure 4:**
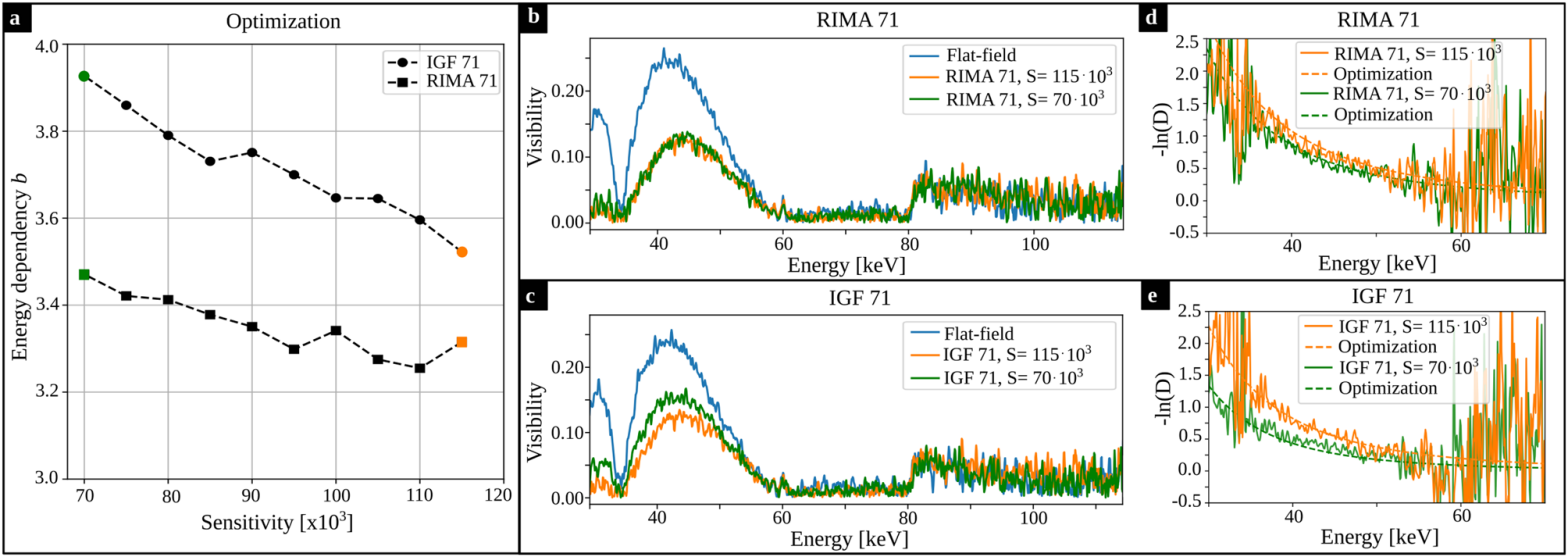
Spectrometer measurements. (**a**) The results from the optimization for all sensitivities investigated (RIMA 71 as squares and IGF 71 as circles). For both materials, the results for the lowest and highest sensitivity (green and orange) are compared to the spectrometer measurements. (**b**,**c**) Measured visibility spectra in the flat-field (blue) and sample measurement for the lowest ($S=70\cdot 10^3$; green) and highest sensitivity ($S=115\cdot 10^3$; orange) for RIMA 71 and IGF 71, respectively. d, e: The measured energy dependent dark-field signal is plotted for both sensitivities in solid lines. The results from the optimization (cf. Eqs. (1) and (5)) are plotted in dashed lines. They were factorized to take into account a sample thickness of 1 cm as used in the spectrometer measurement.

For all sensitivities, the optimized energy dependency of the dark-field signal are plotted in Fig. 4a for IGF 71 and RIMA 71. For both materials, the lowest and highest sensitivity are compared to the spectrometer measurements in the following. The flat-field visibility spectrum measured with the spectrometer is plotted in blue in Fig. 4b,c. The peak at the design energy of 45 keV is lower compared to the one in the simulated visibility spectrum (cf. Fig. 1b), which on the one hand can be a local variation of the visibility over the field-of-view. On the other hand, misalignment and non-gold bridges in the grating lamellae, which support their stability, reduce the visibility due to missing gold structures. Although, the gratings are tilted in beam direction to compensate for this effect, a visibility reduction by bridges can not be completely prevented. However, both simulated and measured visibility spectra agree well in terms of their shape. As mentioned previously, the height in the simulated visibility spectrum was corrected by a constant factor to match the measured and modeled visibility values in the flat-field (cf. Eq. 8 and 9).

The measured visibility for RIMA 71 and IGF 71 is plotted in green and orange for two different sensitivities, $S=70\cdot 10^3$ and $S=115\cdot 10^3$, respectively. Depending on the material and sample position, the visibility spectrum obtained in the sample scan varies. By dividing the visibility from the sample scan by the flat-field visibility for each energy separately, one can obtain the negative logarithmic dark-field signal $-\mathrm{ln}(D)$ as a function of the energy, which is plotted in Fig. 4d,e for RIMA 71 and IGF 71, respectively, for the lowest and highest sensitivity investigated (green: $S=70\cdot 10^3$, orange: $S=115\cdot 10^3$). The dark-field signal calculated with Eqs. (1) and (5) with the results of the optimization are given in dashed lines in Fig. 4d,e. Parameter *a* ([1/mm]) was adapted to fit the spectrometer measurement for 1 cm of the respective material. The energy-dependent dark-field signal depicted in Fig. 4d,e, which are retrieved from the spectrometer measurements, have noise blow 35 keV and above 50 keV. This is due to the low visibility (low signal to noise ratio) at these energy intervals.

## Discussion

The comparison between the optimization approach and the spectrally resolved measurements of the energy dependence of the X-ray dark-field signal shows good agreement for different scattering materials and a large sensitivity range. The energy dependence parameter *b* for all materials decreases for increasing sensitivity. In general, a more quickly falling linear diffusion coefficient is expected for larger structures^19^, which is in agreement with our results (cf. Fig. 4a where a larger *b* was found for IGF 71, which has a larger microstructure). Because the maximum correlation length in this work was much smaller compared to the structure size of the closed-cell foams (and thus, $G(\xi _\mathrm{corr})>0$ for every measurement), the limit for the energy dependence ($\lim _{ G(\xi _\mathrm{corr}) \rightarrow 0} \varepsilon (E) \propto E^{-2}$) was not reached and $b>2$ was valid for all measurements in this work.

For the proposed method a single sample thickness measured with two different photon spectra would in theory be sufficient to optimize for parameter *a* and *b*. However, comparing the expected visibility to the measured visibility for multiple thickness steps increases the reliability of the optimization results.

The X-ray spectra depicted in Fig. 1b show a significant overlap. The source parameters were not optimized to achieve minimum noise for the given imaging task. An additional constraint in grating-based imaging, besides a spectral separation, is the visibility of the system, which has to be sufficiently high for both X-ray spectra. The optimization of the X-ray spectra for grating-based imaging is already demonstrated for the phase-contrast channel^30^ and could also be applied to the dark-field channel.

The detected spectra and the visibility spectrum were simulated in this study. Fabrication inconsistencies, like an inhomogeneous gold height, duty cycle or mismatching periods, as well as the introduction of bridges in the gold lamellae can lead to (local) variations in the visibility spectrum. Especially, the height of the visibility peak is assumed to be decreased in experiments, which is compensated by the correction for consistent flat-field visibility (cf. Eqs. 8 and 9). Besides the visibility spectrum, the photon spectra can also experience changes due to e.g. inhomogeneities of the gratings. In general, alternatives to the simulation of the spectra (both source and visibility spectra) would be either measurements of the spectra with a spectrometer, or estimating the spectra from transmission measurements^31–33^.

The dark-field signal does not increase strictly linearly with the sample thickness. Due to visibility hardening, the dark-field signal flattens out for larger thicknesses^20,34^. This is a polychromatic effect, comparable to beam-hardening in the attenuation channel, and dominant for scattering samples with large thicknesses. Since the thicknesses used for the optimization were small, in the range of a few centimeter, the impact of visibility hardening is minor. Furthermore, the model for the expected visibility in Eq. (6) considers polychromatic effects like visibility hardening properly and thus, is also suitable for larger sample thicknesses. However, the model neglects visibility reduction due to absorption (known as beam hardening induced dark-field signal^35^) at which the X-ray spectrum changes and consequently the overlap between X-ray and visibility spectrum.

The presented approach is therefore only valid for materials with a homogeneous microstructure and weak absorption, regardless of their chemical composition. The lowest transmission was measured for t=30 mm of IGF 71 with the low energy spectrum. We can calculate the change in visibility $\Delta \hat{V}$ induced by attenuation with the density of the material provided in Table 1, its mass attenuation coefficient μ(E) from XMuDat^21^ (chemical formula of polymethacrylimide: $C_{6}H_{7}O_{2}N_{1}$^36^) and the simulated spectrum for the low energy measurement to be,

10 $$\begin{aligned} \Delta \hat{V} = \hat{V}^s - \hat{V}^{s'}, \end{aligned}$$

whereby $\hat{V}^{s'}$ was calculated with Eq. (6) for $t_i=0\,$ mm (no scattering) and the hardened spectrum $D^{s'} (E)$,

11 $$\begin{aligned} D^{s'} (E)=\frac{\Phi ^s (E) \cdot \mathrm{exp} \left[ - \mu (E) \cdot t \right] \cdot \eta (E) \cdot E}{\int \Phi ^s (E) \cdot \mathrm{exp} \left[ - \mu (E) \cdot t \right] \cdot \eta (E) \cdot E \,dE}. \end{aligned}$$

The change in visibility is $\Delta \hat{V}=-0.0006$, which is below the standard deviation of the measured reference visibility ($\sigma _V =0.007$) and hence can be neglected. In general, the model can be extended for the attenuation of the sample when considering strongly absorbing materials with scattering microstructure.

## Conclusion

We proposed an experimental method for the determination of the linear diffusion coefficient based on dual-energy phase-stepping data with a Talbot-Lau interferometer. The method was applied to materials with different microstructure-sizes as well as sensitivities. Although an extension of the model function to absorbing materials and an increase in accuracy through a more accurate determination of the source and visibility spectra are still pending, the method provides access to the quantitative linear diffusion coefficient, which is a prerequisite for upcoming applications of quantitative spectral X-ray dark-field imaging.

## Data Availability

The data sets generated and/or analysed during the current study are available from the corresponding author on reasonable request.
